# Anti-CD antibody microarray for human leukocyte morphology examination allows analyzing rare cell populations and suggesting preliminary diagnosis in leukemia

**DOI:** 10.1038/srep12573

**Published:** 2015-07-27

**Authors:** Alina N. Khvastunova, Sofya A. Kuznetsova, Liubov S. Al-Radi, Alexandra V. Vylegzhanina, Anna O. Zakirova, Olga S. Fedyanina, Alexander V. Filatov, Ivan A. Vorobjev, Fazly Ataullakhanov

**Affiliations:** 1 Centre for Pediatric Hematology, Oncology and Immunology, Moscow, Russia; 2 Centre for Theoretical Problems of Physicochemical Pharmacology RAS, Moscow, Russia; 3 Research Centre for Hematology, Moscow, Russia; 4 Institute of Immunology, Moscow, Russia; 5 A.N. Belozersky Institute and Department of Cell Biology and Histology, Faculty of Biology, M.V. Lomonosov Moscow State University, Moscow, Russia

## Abstract

We describe a method for leukocyte sorting by a microarray of anti-cluster-of-differentiation (anti-CD) antibodies and for preparation of the bound cells for morphological or cytochemical examination. The procedure results in a “sorted” smear with cells positive for certain surface antigens localised in predefined areas. The morphology and cytochemistry of the microarray-captured normal and neoplastic peripheral blood mononuclear cells are identical to the same characteristics in a smear. The microarray permits to determine the proportions of cells positive for the CD antigens on the microarray panel with high correlation with flow cytometry. Using the anti-CD microarray we show that normal granular lymphocytes and lymphocytes with radial segmentation of the nuclei are positive for CD3, CD8, CD16 or CD56 but not for CD4 or CD19. We also show that the described technique permits to obtain a pure leukemic cell population or to separate two leukemic cell populations on different antibody spots and to study their morphology or cytochemistry directly on the microarray. In cases of leukemias/lymphomas when circulating neoplastic cells are morphologically distinct, preliminary diagnosis can be suggested from full analysis of cell morphology, cytochemistry and their binding pattern on the microarray.

Matching the morphology with immunophenotype for individual leukocytes is a major issue in diagnostics of leukemia and lymphoma in cases of aberrant immunophenotypes or atypical morphologies as well as in research. The absence of a method for simultaneous cluster of differentiation (CD) surface antigen detection and full leukocyte morphology analysis hinders the characterisation of rare morphological subtypes of normal and atypical leukocytes. Immunofluorescent staining of the smear cannot be combined with staining for morphology due to the high non-specific fluorescence of the dyes used in the morphology stain. From the three possible ways to overcome this, simultaneous staining for morphology analysis and for CD antigens (by immunocytochemistry^1^ or image flow cytometry^2^), sorting by morphology^3^,^4^,^5^ and sorting by surface CD antigens, the first two have limited applicability or produce low-quality results. The third approach can be realised using a leukocyte-binding antibody microarray.

Antibody microarrays^6^ were first applied for binding of whole cells by Chang^7^. Anti-CD antibody or aptamer microarrays for leukocyte “panning” by their surface antigens were developed by several groups^8^,^9^,^10^,^11^,^12^,^13^,^14^. However all these works focused on determination of relative content of the cells positive for certain CD antigens in analysed samples, the information conventionally obtained by flow cytometry. The morphology of the microarray-bound cells was not assessed.

Here we describe an anti-CD antibody microarray on a transparent support for leukocyte sorting and a method for preparation of the microarray-bound cells for high-resolution morphology analysis (Fig. 1). We show that the microarray works as a “cell-sorted” smear as the cell binding is highly specific, the microarray-captured peripheral blood mononuclear cells are morphologically identical to the same cells in a smear and are suitable for other standard smear-oriented techniques such as cytochemistry. The microarray permits to determine the proportions of cells positive for any CD antigen on the microarray panel with high correlation with flow cytometry. We prove that the microarray can be used to determine the immunophenotype corresponding to the cells of certain morphology by analysing the percentage of these cells among the leukocytes captured by different anti-CD antibodies. Using this approach we show that normal peripheral blood mononuclear cells with granular lymphocyte morphology and with radial segmentation of the nuclei are positive for CD3, CD8, CD16 or CD56 but never for CD4 or CD19. We finally demonstrate that the microarray can be used to obtain a pure leukemic cell population or to separate two leukemic cell populations on different antibody spots ready for morphological or cytochemical examination directly on the microarray and show the advantages of this pure population analysis in leukemia diagnosis.

## Results

### Optimization of the microarray preparation and leukocyte panning procedure

The anti-CD capture antibodies were immobilised on a transparent polyvinylchloride slide by adsorption during overnight incubation at 4 ^°^C. Figure 2A shows the distribution of the bound cell density across the antibody spot for different adsorption protocols. The antibody incubation overnight at 4 ^°^C followed by washing and blocking in 1% BSA solution resulted in 10-fold smaller on-the-spot cell density compared to the antibody incubation overnight at 4 ^°^C followed by drying at the same temperature, washing and blocking. Drying at room temperature resulted in a non-uniform cell distribution across the spot with high cell density on the outside and lower cell density in the middle of the spot. This effect is caused by the non-uniform antibody distribution often seen in protein microarrays (the “donut structure”^15^). Antibody drying at 4 ^°^C resulted in a uniform on-the-spot cell distribution with cell density of 7000–8000 cells/mm^2^. This number is close to the theoretical limit of the bound cell density of 6945–8265 cells/mm^2^ estimated for round cells with 11–12 micrometer diameters. Figure 2B,D show the on-the-spot bound cell density dependence upon the antibody concentration in the spotting solution for anti-CD45, anti-CD2, anti-CD3, anti-CD4, anti-CD8 and anti-CD19. The bound cell density saturates at 10–50 microgram/ml antibody concentrations. These concentrations are lower than the concentrations used by Belov *et al.*^8^ and Zhu *et al.*^14^ as the microarrays described in these papers use nitrocellulose or hydrogel-covered glass with higher antibody-binding density but lower antibody accessibility for the binding cells.

Figure 2C shows the overlay of the phase contrast and fluorescence pictures of the microarray with bound PBMC stained with FITC-labelled anti-mouse IgG. The cell binding outside the microarray spot with immobilised IgG is negligible (below 20 cells/mm^2^). However, when the PBMC suspension was incubated with the microarray at room temperature, we have noted a significant amount of nonspecific monocyte binding on the antibody spot (Fig. 2E). This binding could not be diminished by addition of 10% fetal bovine serum or 6% BSA to the cell incubation buffer (results not shown). As the nonspecific monocyte binding at room temperature occurred on the antibody spots but not in the space between them, we supposed this binding to be linked to the antibody-covered surface and to have the same mechanism as the phagocytosis of opsonized particles by peripheral blood monocytes. Monocyte phagocytosis temperature dependence has a threshold at 18 ^°^C as at lower temperatures monocytes fail to ingest IgG-covered particles^16^. In our experiments nonspecific on-the-spot monocyte binding does not exceed 15 cells/mm^2^ when the PBMC are incubated with the microarray at 4 ^°^C.

The bound cell density depends upon the cell concentration in the analysed suspension. Figure 2F shows the density of the cells bound to the spots with immobilised anti-CD3, anti-CD19 and anti-CD45 antibodies when different concentrations of the same mononuclear cell suspension were analysed with the microarray. The bound cell density saturated at 7·10^6^ cells/ml in analysed suspension. It is of note that the ratio of the densities of anti-CD3, anti-CD19 and anti-CD45-captured cells does not significantly change with the cell concentration in the analysed suspension, as the titrations in Fig. 2F could be global fitted with y = a·(1 – exp(–x/x_0_)) function with the same x_0_ for anti-CD3, anti-CD19 and anti-CD45. This fact permits to compare the ratio of microarray-bound cell densities for all the antibodies for wide range of cell densities in analysed cell suspension.

### Cell binding to the microarray is highly specific

The specificity of the on-the-spot lymphocyte binding was determined for the main lymphocyte groups (T helper cells, cytotoxic T cells and B cells) by incubating the PBMC prestained with fluorochrome-conjugated antibodies against CD3, CD4, CD8 and CD19 with the microarray and counting the amount of fluorochrome-labelled cells captured by anti-CD3, anti-CD4, anti-CD8, anti-CD19 and anti-CD45 spots on the microarray. The fraction of the PBMC captured by immobilised anti-CD3, anti-CD4, anti-CD8 or anti-CD19 that stained positively for the same antigens varied from 93 ± 7% to 98 ± 2%. Nonspecific binding (CD19-positive cells, captured on the microarray by anti-CD3, anti-CD4 or anti-CD8; CD4-positive cells, captured by anti-CD8 or vice versa) did not exceed 2 ± 2% (Fig. 3, Table 1).

### Normalised density of antibody-captured cells on the microarray matches the percentage of the cells positive for the corresponding antigen determined by flow cytometry

**Figure 1:**
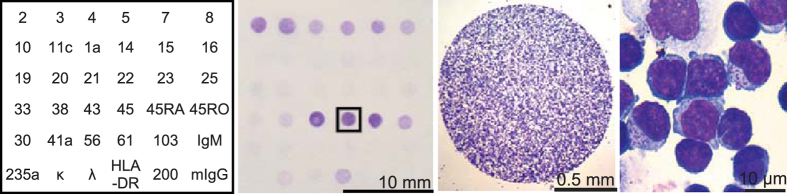
The anti-CD antibody microarray working principle. (Left) The “map” of the microarray with numbers indicating the spots of mouse IgG against corresponding CD antigens; mIgG indicates the negative control; (middle left) the whole microarray with captured normal PBMC after May-Grünwald-Giemsa staining; (middle right and right) the anti-CD45-bound normal PBMC at different magnifications.

Figure 1 (middle left) shows that the cell densities of normal PBMC captured by the microarray differ for different antibodies. This density difference reflects the difference in the percentage of PBMC positive for different CD antigens if the cells are incubated with the microarray in non-mixing conditions as illustrated by Fig. 4A. Thus in non-mixing conditions the percentage of the cells positive for a CD can be estimated as the density of the cells captured by the corresponding antibody normalised by the number of cells captured by the anti-CD45 (positive control) spot on the same microarray. The percentage of cells positive for CD3, CD4, CD8 and CD19 from all CD45-positive cells determined for the same PBMC suspension by flow cytometry for 14 healthy donors was in accord with the percentages calculated from the density of the microarray-bound cells as described above with correlation coefficient between the data obtained by the microarray and with flow cytometry R > 0.89 (Fig. 4B). Medians and 95% confidence intervals for the PBMC percentage positive for several CD antigens obtained by the analysis on the microarray (Figure S1) match the published reference ranges derived from flow cytometry^17^,^18^,^19^,^20^,^21^,^22^.

### Morphology of the cells captured by the anti-CD microarray is similar to their morphology in a standard blood smear

Since the smear is considered as a gold standard for morphology examination, the main challenge of the work is to make the microarray-captured cells morphologically identical to those in a blood smear. During the blood smear preparation the cells are (i) flattened by the surface tension during the spreading procedure to make the inner leukocyte structures more visible and (ii) quickly dried in isotonic conditions (due to the thinness of the layer) preserving the cell membranes intact. We imitate these two steps by covering the microarray-captured cells with fetal calf serum and drying them by rotation around the axis normal to the microarray surface.

Figure 5 shows the main classes of PBMC morphology in a smear (Fig. 5A–E) and on the microarray (Fig. 5F–O)^1^,^23^. Comparison of PBMC observed on the microarray (anti-CD45 spot) and in a smear shows that the cell areas of lymphocytes and monocytes vary within the same limits and have close median values (Fig. 5P). As the morphology of the microarray-captured cells is similar to the smear, the morphological classification of PBMC developed for the blood smear can be applied to the cells captured by the microarray.

### Rare morphological types of normal lymphocytes are determined and classified as B-, T- and NK-cells

#### Granular lymphocytes are predominantly captured by anti-CD8, anti-CD16 and anti-CD56

Table 2 shows average percentages of small lymphocytes, large lymphocytes, granular lymphocytes and monocytes among the PMBC captured by the antibodies against the main lineage-specific CD antigens. Small lymphocytes constituted the majority of PMBC captured by all antibodies except anti-CD16 and anti-CD56. Large lymphocytes (LL) without cytoplasmic granulation accounted for 1.9 ± 0.2% to 4.7 ± 0.4% of cells captured by the antibodies against the CD antigens, presented in Table 2. The LL percentage was significantly higher for CD19+ cells than for CD3+ cells (p = 10^−4^ by double-sided paired t-test) in line with the results of Strokotov *et al.*^24^. Monocytes were only captured by the antibodies against CD14, CD4, CD16 and CD45, the antigens that they are known to express.

The main difference in the morphological cell type distribution between the anti-CD antibodies is observed for the large granular lymphocytes. No GL are captured by anti-CD19 showing that none of the GL is a B-cell. No GL are captured by anti-CD4. However, about a quarter of all anti-CD8-captured as well as half of anti-CD16 and anti-CD56-captured lymphocytes are granular (see Table 2), suggesting that most of GL have an immunophenotype typical for cytotoxic T-cells or NK cells^25^.

#### Lymphocytes with lobed nuclei are either NK- or CD8+ T-cells; nuclei with single clefts are typical for the B-cells

Lymphocytes with lobed nuclei^26^ (a subset of large lymphocytes that have radially segmented nuclei with two or more clefts at least half a nuclear radius deep) and granular lymphocytes with lobed nuclei are rare morphological types that are nevertheless observed in every healthy donor. They account for up to 1% of PMBC captured by anti-CD45. This compares well to the observation that about 0.5 ± 1% of normal peripheral blood lymphocytes have minor nuclear indentations with nuclear contour index (NCI, determined as a nuclear circumference divided by the square root of the nuclear area) of 4.1–5.9^27^. Fig. 5Q shows that NCI index for the lymphocytes with lobed nuclei varies from 4.2 to 5 with a median of 4.4 while median NCI for non-lobed nuclei is 3.75.

Lymphocytes with lobed nuclei and granular lymphocytes with lobed nuclei accounted for 1.4 ± 0.2 and 1.1 ± 0.3% of PMBC captured by anti-CD2 antibody and 0.8 ± 0.1% and 0.6 ± 0.1% of PBMC captured by anti-CD3 respectively indicating their T- or NK-cell origin. No granular or agranular lymphocytes with lobed nuclei were captured by anti-CD19. The highest percentage of LLN and GLLN was observed for the PBMC captured by anti-CD56 (3.5 ± 2% and 4.7 ± 1% respectively). About 1.5% of anti-CD8- and 2% of anti-CD16- captured PMBC were LLN and the same amount - GLLN. Our data (summarised in Table 2) suggest that lymphocytes with a lobed nucleus are either NK- or CD8+ T-cells.

Binuclear lymphocytes were observed in 7 donors out of 40 and were captured only by anti-CD19, anti-CD20 or anti-CD22 supporting the supposition that deep single clefts in nuclei or binuclear appearance are typical for the B-cells and have a different underlying mechanism from the radial segmentation causing the “clover-leaf” nuclei^28^.

### Peripheral blood of patients with leukemias and lymphomas studied by the anti-CD antibody microarray

To test the applicability of the anti-CD microarray to leukemia diagnosis we have studied the PBMC of 77 patients with different leukemias and lymphomas: chronic lymphocytic leukemia (CLL, 37 patients), hairy cell leukemia (HCL, 22 patients), splenic marginal zone lymphoma (SMZL, 7 patients), mantle cell lymphoma (MCL, 2 patients), follicular lymphoma (FL, 1 patient), 5 patients with multiple myeloma (MM), 2 patients with large granular lymphocytic (LGL) leukemia and one patient with acute myeloid leukemia (AML M2). The control group contained 55 healthy donors. For each patient we determined (i) the density of the PBMC captured by every antibody on the microarray normalised to the density of anti-CD45-captured cells and (ii) the antibodies that captured the neoplastic cells (defined by their morphology) in order to elucidate their immunophenotype.

The normalised densities of PBMC from the patients with B-cell malignancies CLL, HCL, SMZL, MCL, FL captured by the antibodies against the T-cell markers CD2, CD3, CD4, CD7, CD8 were significantly lower and by the antibodies against the B-cell markers CD19, CD20 and CD22 - significantly higher than for normal controls (see Fig. 6A–C for CLL, HCL and SMZL). The density of anti-CD5-captured cells was lower than controls for FL, HCL and SMZL (Fig. 6B–C) but not for CLL (Fig. 6A) and MCL. There was also a dramatic increase in the densities of anti-CD23-captured cells for CLL and anti-CD25-captured cells in HCL. Highly variable amounts of anti-CD11c and anti-CD103-captured cells appeared in HCL patients (Fig. 6B) and high amount of anti-CD10-captured cells in FL patient (not shown) that were absent in controls. These changes reflect the appearance of leukemic CD19+, CD20+, CD22+ B-cells with coexpression of CD5 and CD23 for CLL, CD11c, CD25 and CD103 for HCL, CD5 for MCL and CD10 for FL and are in good agreement with literature^29^,^30^. The normalised densities of microarray-captured PBMC from the patients with MM and LGL leukemia did not differ significantly from normal controls. The pattern of the microarray binding densities for the PBMC from the studied patients differs from the corresponding flow-cytometric pattern in the highly variable densities of CD20 and CD22 positive cells for CLL. This can be accounted for by a significant decrease of CD20 and CD22 expression on CLL cells compared to normal B lymphocytes^29^,^31^,^32^ that results in insufficiently strong lymphocyte binding to the microarray and cell elimination during the washing step. However, this relative drop in the densities of anti-CD22 and CD20-captured cells is highly specific, as the decrease in CD22 and especially in CD20 expression per cell marks CLL compared to all other chronic B-cell leukemias^33^.

The morphology of the microarray-captured leukemic cells was identical to their morphology in smears and in perfect agreement with literature for CLL^34^,^35^, SMZL^36^,^37^, MCL, FL, MM, AML, LGL leukemia^29^, (Figure S3A–H) and HCL^29^ (Fig. 6D–F).

### Cytochemistry

Detection of the enzyme activity characteristic of certain leukocyte lineages (cytochemistry) is used in diagnosis to identify the lineage of the neoplastic cells. We have found that the standard protocols for Sudan black B stain, detection of α-naphtyl acetate esterase used in acute myeloblastic leukemia diagnosis (Figure S2) and tartrate-resistant acid phosphatase used in differential diagnosis of hairy cell leukemia can be applied to microarray-bound cells. Figure 6G–I show identical tartrate-resistant acid phosphatase (TRAP) activity detected in the peripheral blood lymphocytes from the same patient with HCL in a smear (Fig. 6G) and on the microarray (Fig. 6H–I).

### Diagnostic applications of anti-CD microarray

The close correlation between the PBMC immunophenotype, morphology and cytochemistry determined by the anti-CD microarray and by standard diagnostic methods suggests a strategy for the microarray application in leukemia diagnosis. The change in normalised cell binding densities compared to normal controls can suggest a presence of neoplastic cell population. In cases when pathologic cells are morphologically and/or cytochemically distinct, the anti-CD antibody microarray permits to determine their percentage and immunophenotype by analysing the relative amount of these cells captured by the antibodies against all the CD antigens. The results of such analysis of neoplastic PBMC for the patients with leukemias and lymphomas we have studied are presented in Tables S1–S3 and agree with flow cytometry results for the same patients including expression of CD2 in HCL, CD2 and CD11c in CLL, CD56 in MM (not shown). The amount of hairy cells determined morphologically on the microarray varied from 20 to 97 of all anti-CD19-captured cells and 2 to 80% of all lymphocytes and was in good agreement with the percentages of cells with CD19/CD103 and CD19/CD11c coexpression determined for the peripheral blood of the same patients by flow cytometry. We have also shown the possibility of microarray analysis for leukemic cells from pleural effusion and homogenised solid biopsy specimens (spleen, lymph node and gastric samples) (Fig. S3I–L, Tables S2, S3). Morphology and immunophenotype of leukemic cells in these samples were confirmed by standard diagnostic methods.

The microarray spots with antibodies against the antigens specific for neoplastic cells contain their pure or highly concentrated population (e.g. only hairy cells are captured by anti-CD11c and CD103 (Fig. 6E,H)) available for morphological, cytochemical or any other analysis performed in a smear such as fluorescent *in situ* hybridization. It also increases the sensitivity of morphological analysis: in case of HCL when the patients are usually leukopenic^29^ this approach permits to detect hairy cells present in peripheral blood in concentration as low as 50 cells/μl which is below the limit of detection in a smear.

Combined analysis of the pathologic cells’ immunophenotype, morphology and cytochemistry on the microarray permits to arrive at preliminary diagnosis and can be used in cases of any controversies between morphology, cytochemistry and immunophenotyping. Examples of such analysis are given in supplementary information. Figure S4A–B and case 1 in data S1 illustrate a case of differential diagnosis between HCL and SMZL. HCL was suggested based on peripheral blood and bone marrow aspirate analysis and the trephine bioptate immunohistochemistry, while the flow cytometry data was more consistent with SMZL. The patient was diagnosed with SMZL with circulating villous lymphocytes based on flow cytometry data combined with tartrate-resistant acid phosphatase (TRAP) negativity of pathologic lymphocytes and the presence of clonal IgM secretion. The analysis of the PBMC morphology of this patient on the microarray shows the increased amount of B-cells while the amount of CD11c and CD103-positive cells does not differ from the healthy controls. There are two morphological types of neoplastic B-lymphocytes present (Figure S4A–B), both being positive for CD19, CD20, CD22, CD25 but not CD23, CD10, CD11c or CD103. This microarray-derived immunophenotype of the pathologic cells together with the heterogeneity of the leukemic cells and the morphologic characteristics of their subgroups that could not be observed in peripheral blood smears due to their low content (6% of total PBMC) are incompatible with HCL and highly characteristic of SMZL^36^,^37^.

Figure S4C–E and case 2 in data S1 shows the separation of two leukemic cell lines in acute bilineal leukemia (T-myelo) on the microarray. This separation of two morphologically different blast lines permits to diagnose acute bilineal leukemia from full analysis of leukemic cell morphology and patterns of their binding to the microarray as well as to study the blasts of both lines separately by other methods such as fluorescence *in situ* hybridization and potentially facilitates the control of both cell lines during treatment.

## Discussion

The microarray of anti-CD antibodies permits to isolate pure leukocyte subsets without the use of magnetic beads or fluorescence activated cell sorting. The normalised density of the cells captured by an anti-CD antibody gives the percentage of the cells positive for this CD in the analysed PBMC suspension and the results are in excellent agreement with flow cytometry (Fig. 4B). Although the possibility of using the density of the microarray-captured PBMC as a measure for the percentage of the cells positive for certain antigens has been suggested before^8^,^14^, here we give the first quantitative comparison of the normalised cell density to flow cytometry. The patterns of the binding densities of the anti-CD-captured PBMC for chronic B-lymphocytic leukemia, hairy cell leukemia and splenic marginal zone lymphoma patients clearly differ both from those for normal PBMC and from each other and agree well with the reported immunophenotypes of corresponding neoplastic cells.

The drying procedure flattens the cell on the microarray surface in a way similar to the smear preparation resulting in a “sorted cell smear” with cells positive for a certain surface CD antigen localised in a predetermined area and permitting to apply any standard smear-oriented technique (including morphological and cytochemical staining) to the microarray-captured cells. The closeness of the PBMC morphology on the microarray and in a smear (Fig. 5) permits to apply the established morphological classification of PBMC to the cells on the microarray. The microarray preserves the ratio between different PBMC classes where it can be verified. Large and large granular lymphocytes account for 9% (3% to 20%) of all the lymphocytes captured by anti-CD45 in good agreement with the data on total large lymphocyte percentage in peripheral blood smears of about 10%^1^. On top of this, the PBMC isolation and high cell binding density on the microarray permit to analyse up to 100000 cells in total and to find rare cell types such as lymphocytes with lobed nuclei or binuclear lymphocytes while the cell panning by their surface CD antigens classifies them as B-, T- or NK cells.

Most of the data on the immunophenotype of the granular lymphocytes (GL) comes from the immunocytochemistry experiments on GL-enriched lymphocyte fraction after PBMC fractionation in 7-step Percoll gradient^5^,^4^,^38^ and thus cannot account for all granular lymphocytes. This data as well as the studies of CD56-positive lymphocytes^39^ indicate that most granular lymphocytes are NK cells and most but not all NK cells have large granular lymphocyte morphology although some granular lymphocytes (5% or 10–20% depending on the author) are T-cells with predominantly CD3+CD8+ immunophenotype. Another evidence for the nature of GL comes from the data on large granular lymphocytic leukemia^40^ where the neoplastic cells usually have either T-cell (CD3+CD8+) or NK-cell (CD3-CD56+) immunophenotype. Here we show directly for the first time that normal PBMC with granular lymphocyte morphology are positive for CD3, CD8, CD16 or CD56 and negative for CD4 or CD19.

The early studies of normal lymphocytes with clefted and radially segmented nuclei^26^,^41^,^27^ together with the data on the neoplasms where the leukemic lymphocytes obtain single nuclear clefts (FL, MCL)^29^ or “clover-leaf” nuclei (adult T-cell leukemia/lymphoma)^29^,^28^ suggest that the radially segmented nuclei are only observed in T-cells while single clefts are specific for B-cells. Our results show that the lymphocytes with lobed nuclei isolated from the peripheral blood of healthy individuals and captured by the anti-CD antibody microarray are either NK- or CD8+ but not CD4+ T-cells in contrast with the lymphocytes with mitogen-induced lobulation of the nuclei^27^.

The simple technique described here can be used to study the correlation between the morphology and immunophenotype for normal and pathological leukocytes, the mechanisms of the formation of certain morphological features such as the cytoplasmic villi or radial segmentation of the nucleus. It can be useful for diagnostics in cases of low leukemic cell content - to concentrate the neoplastic cells, in presence of two neoplasms - to separate the cells corresponding to each neoplasm or in cases of dubious morphology and/or aberrant immunophenotype – to match the morphology to the immunophenotype to arrive at the correct diagnosis. The possibility to suggest a preliminary diagnosis from full analysis of cell morphology, cytochemistry and binding pattern on the microarray can also prove invaluable for leukemia diagnosis in resource-poor countries.

## Methods

### Antibodies used in the microarray fabrication

Anti-CD1a (HI149, eBioscience), anti-CD2 (LT2, Sorbent LTD), anti-CD3 (TB3, Sorbent LTD), anti-CD4 (OKT4, eBioscience), anti-CD5 (LT1, Sorbent LTD), anti-CD7 (LT7, Sorbent LTD), anti-CD8 (OKT8, eBiosciences), anti-CD10 (LT10, Sorbent LTD), anti-CD11c (3.9, eBiosciences), anti-CD14 (61D3, eBioscience), CD15 (HI98, eBioscience) anti-CD16 (LNK16, Sorbent LTD), anti-CD19 (LT19, Sorbent LTD), anti-CD20 (LT20, Sorbent LTD), anti-CD21 (LT21, Sorbent LTD), anti-CD22 (LT22, Sorbent LTD), anti-CD23 (LT-4F1, Sorbent LTD), anti-CD25 (BC96, eBioscience), anti-CD33 (WM53, eBioscience), anti-CD38 (LT38, Sorbent LTD), anti- CD41a (HIP8, eBioscience), anti-CD43 (eBio84-3C1, eBioscience), anti-CD45 (H130, eBioscience), anti-CD45RA (HI100, eBioscience), anti-CD45RO (UCHL1, eBioscience), anti-CD56 (LT56, Sorbent LTD), anti-CD61 (VI-PL2, eBioscience), anti-CD64 (10.1, eBioscience) anti-CD103 (B-Ly7, eBioscience), anti-HLA-DR (LN3, eBioscience), anti-human IgG kappa light chain (TB28-2, eBioscience), anti-human IgG lambda light chain (1-155-2, eBioscience), anti-human IgM (Sorbent LTD), anti-CD117 (YB5.B8, eBioscience), mouse IgG1 K isotype control (P3.6.2.8.1, eBioscience).

### Microarray fabrication

Anti-CD antibodies in carbonate-bicarbonate buffer (pH 9.2) were spotted manually with a hand-held pipette in 1 μl spots on unprepared plastic coverslips (Fisher Scientific), incubated overnight at 4 ^°^C in a wet chamber and dried at 4 ^°^C. The dried coverslips were blocked in 6% bovine serum albumin (BSA) in phosphate buffered saline (PBS) pH 7.4) for 1 hour, washed with water, dried and stored with desiccator at 4 ^°^C until use. Prior to use the microarrays were briefly incubated in 1% BSA in PBS. The antibody concentration in the spotting solution was 0.25–0.75 μg/ml.

### Leukocyte sorting by the microarray

**Figure 2:**
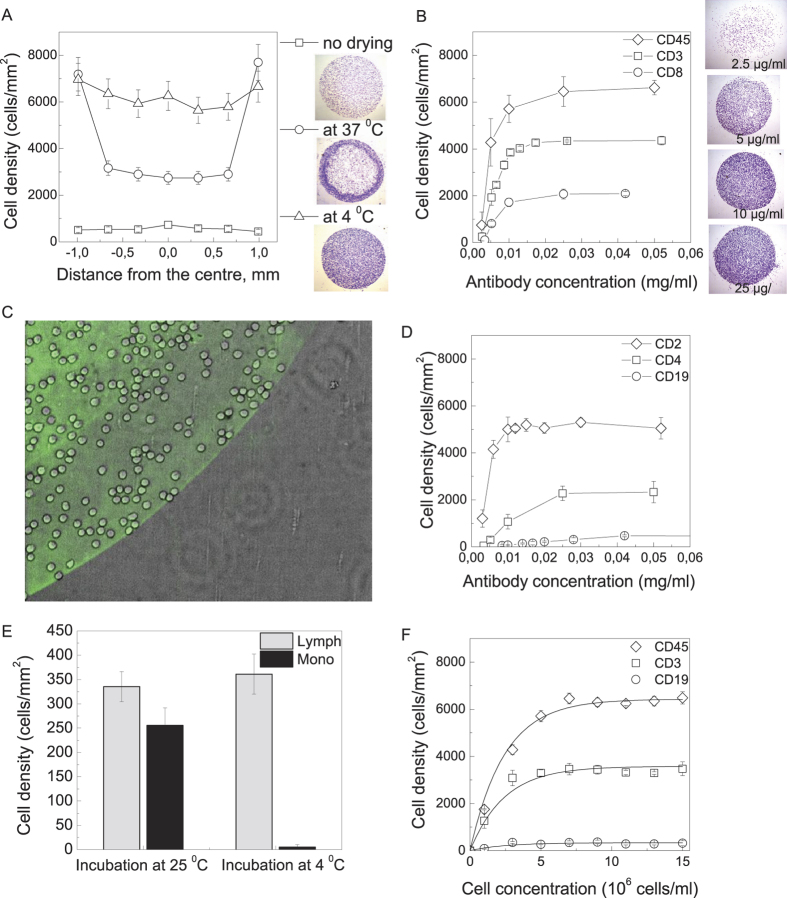
Optimization of microarray preparation and cell-panning protocols. (**A**) The edge-to-edge plot of bound cell density for anti-CD45 antibody, spotted onto the plastic coverslip at 0.25 mg/ml and the brightfield images of the mononuclear cells captured on the anti-CD45 antibody spots for different immobilization methods: overnight incubation at 4 ^°^C, no drying (top right), drying at 37 ^°^C (middle right), drying at 4 ^°^C (bottom right); (**B,D**) The density of the captured peripheral blood mononuclear cell for different concentrations of anti-CD45, anti-CD3, anti-CD8 (**B**) and anti-CD2, anti-CD4, and anti-CD19 (**D**) immobilised by overnight incubation at 4 ^°^C with subsequent drying at 4 ^°^C. On top of (B): the brightfield images of the mononuclear cells captured on the anti-CD45 antibody spots for different antibody concentrations. PMBC concentration 8·10^6^/ml; (**C**) Overlay of DIC (grey) and fluorescent (green) pictures of PBMC, captured on anti-CD4, stained by FITC-labelled anti-mouse I gG. Note that no cells bind outside the green area that indicates immobilised antibodies; (**E**) The density of lymphocytes and monocytes captured by anti-CD19 antibodies for different incubation conditions. PMBC at 8·10^6^ cells/ml concentration were incubated on the microarray for 30 min at room temperature and at 4 ^°^C. Monocytes were determined morphologically at ×1000 magnification after May-Grünwald-Giemsa staining; (**F**) The density of PBMC captured by anti-CD45, anti-CD3 and anti-CD19 from cell suspensions taken at different concentrations. The dots represent the experimental data, the curves result from a global fit with y_i_ = a_i_·(1 – exp(–x/x_0_)) function. The fitting parameters are: x_0_ = 2.4 ± 0.2, a_1_(CD45) = 6400 ± 100, a_2_(CD3) = 3580 ± 90, a_3_(CD19) = 330 ± 80.

**Figure 3:**
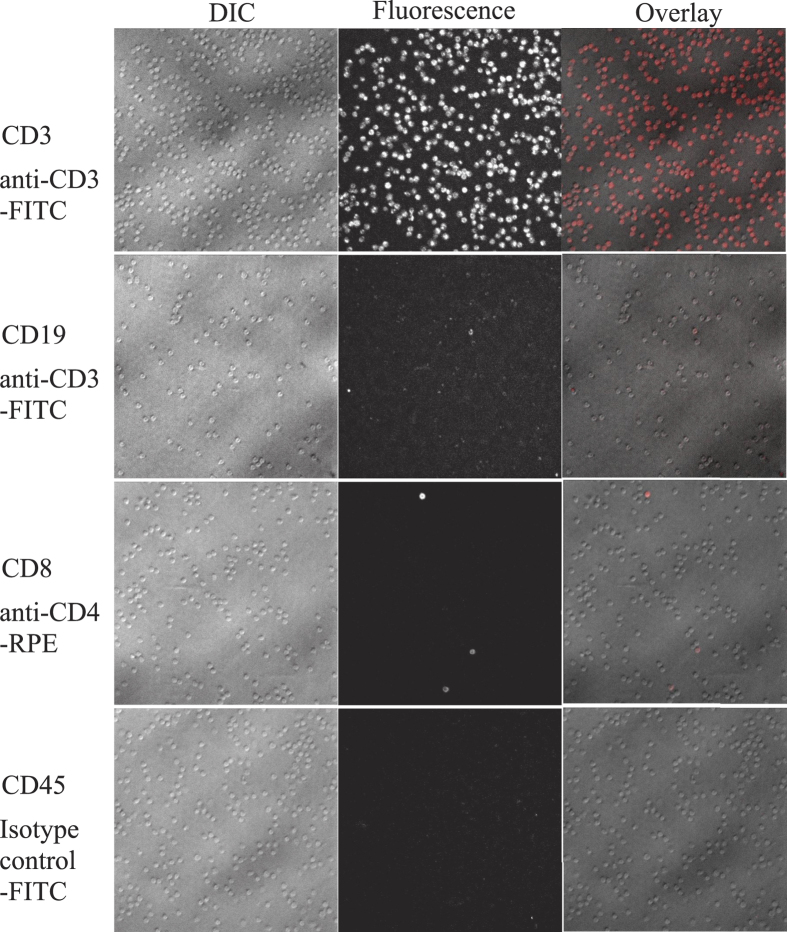
Specificity controls for the lymphocyte panning by the microarray. DIC, fluorescence and overlay pictures of anti-CD3-FITC-labelled cells, captured by anti-CD3 antibody on the microarray, anti-CD3-FITC-labelled cells, captured by anti-CD19 antibody on the microarray, anti-CD4-RPE-labelled cells, captured by anti-CD8 antibody on the microarray, isotype control mouse IgG-FITC-labelled cells, captured by anti-CD45 antibody on the microarray. Original magnification ×400.

**Figure 4:**
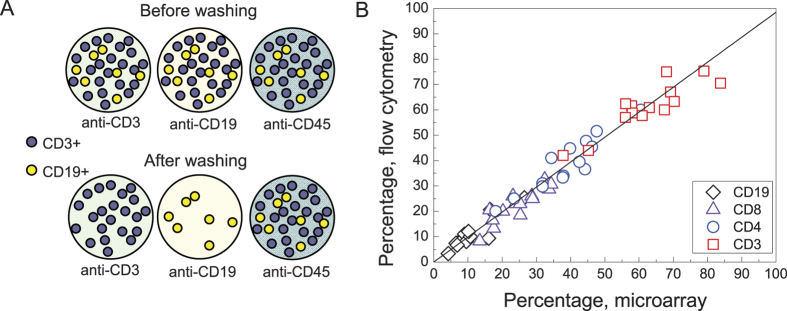
Microarray application to immunophenotype determination. (**A**) A scheme explaining the proportionality between the density of the anti-CD-captured cells and the content of the cells positive for the same CD in the analysed cell suspension; (**B**) Comparison of flow cytometric and microarray analysis of the same mononuclear cell suspension isolated from the peripheral blood of 14 healthy donors. Y axis – number of PBMC, positive for CD3, CD4, CD8 and CD19, normalised to the number of CD45+ cells, determined by flow cytometry. X axis – the density of PBMC captured by anti-CD3, CD4, CD8 and CD19 antibodies from the same cell suspension, normalised to the cell density on anti-CD45. The correlation coefficients between the microarray and flow cytometry data are R = 0.89 for CD3, R = 0.93 for CD4, R = 0.89 for CD8 and 0.91 for CD19. The best linear fit (y = k·x) gives k = 0.99 ± 0.2.

**Figure 5:**
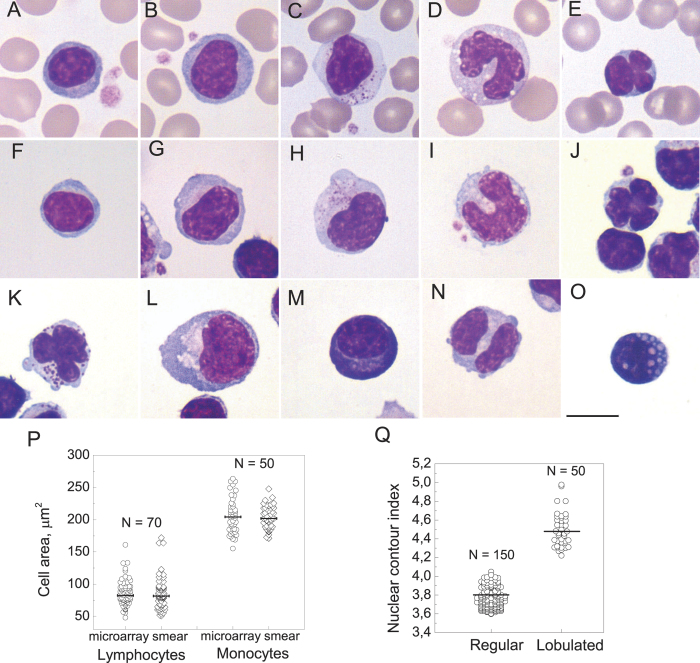
Morphological classification of mononuclear cells in a blood smear. (**A–C**) and captured on the microarray from the PBMC suspension from healthy donors (**F–O**). (**A,F**) small lymphocyte up to 12 micrometres in diameter with nuclear-cytoplasmic (NC) ratio above 3:1 and no visible cytoplasmic granules; (**B,G**) large lymphocyte 13 –15 micrometres in diameter with nuclear-cytoplasmic (NC) ratio 3:1 - 2:1 with pale cytoplasm without visible granules; (**C,H**) granular lymphocyte, lymphocyte of any size with cytoplasmic granules; (**D,I**) monocyte; (**E,J**) lymphocyte with a lobed nucleus, having two or more nuclear clefts at least half a nuclear radius deep; (**K**) granular lymphocyte with a lobed nucleus; (**L**) reactive lymphocyte 15–20 micrometres in diameter with NC ratio 2:1 - 1:1 and light blue to deep blue cytoplasm with peripheral accentuation); (**M**) plasma cell; (**N**) binuclear lymphocyte; (**O**) Mott cell. The scale bar represents 10 μm. May-Grünwald-Giemsa stain, original magnification ×1000. (**P**) The cell area distributions for lymphocytes and monocytes on the microarray and in a blood smear. The lines represent the median values with N = 70 for the lymphocytes and N = 50 for the monocytes. The cell area distributions do not differ by Mann-Whitney criteria with p = 0.6 for the lymphocytes and p = 0.8 for the monocytes; (**Q**) The nuclear index distribution for 150 lymphocytes with non-lobed nuclei and 50 lymphocytes with lobed nuclei.

**Figure 6:**
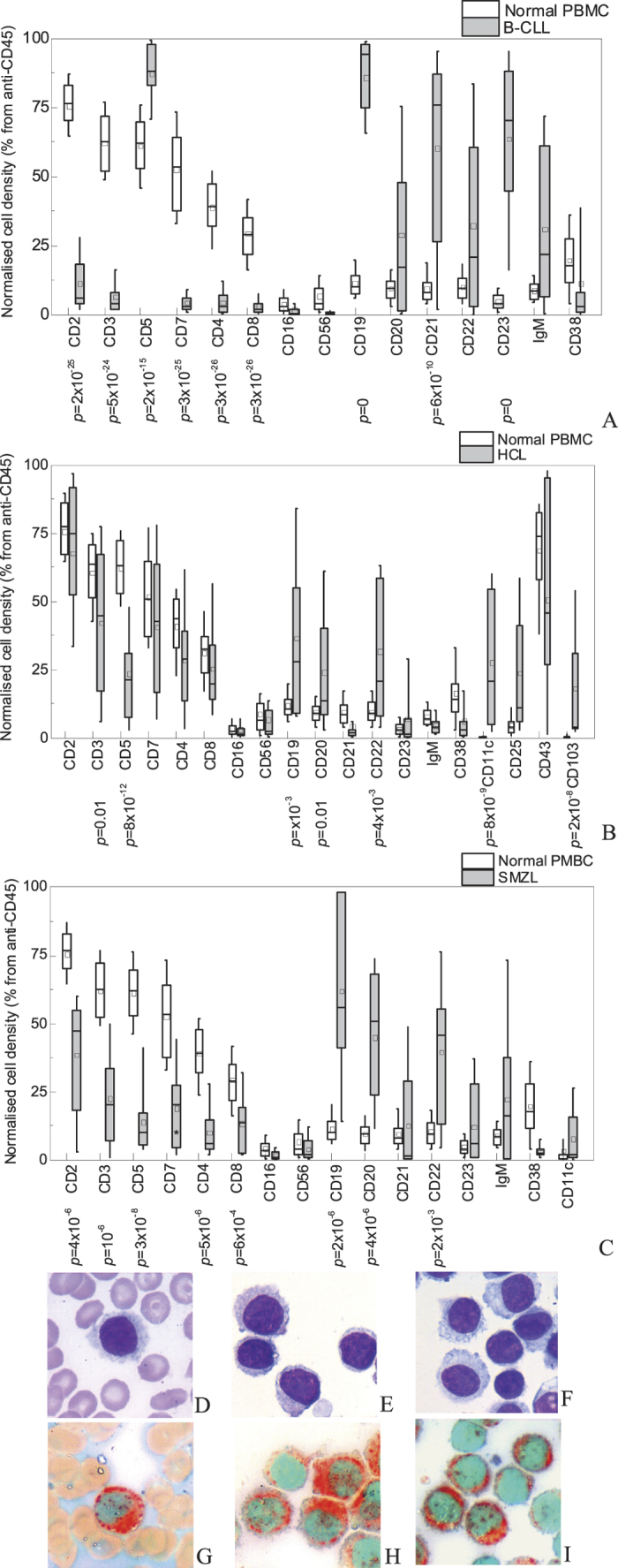
The microarray-captured mononuclear cells from the peripheral blood of the patients with chronic B-cell leukemias and lymphomas. (**A–C**) The density of cells, captured by different anti-CD, normalised to the cell density on anti-CD45, for PBMC from 55 healthy donors (white boxes) and PBMC isolated from 22 patients with chronic B-lymphocytic leukemia (A, grey boxes), 20 patients with hairy cell leukemia (B, grey boxes) and 7 patients with splenic marginal zone lymphoma (C, grey boxes). The boxes indicate the 25%–75% confidence interval, the bars – 10%–90% confidence interval, the middle line indicate the median value, the square – the average value. The *p* values calculated according to Mann-Whitney U-criteria are given below the x-axis when below 0.05; (**D–I**) morphology after May-Grünwald-Giemsa stain (**D–F**) and cytochemistry test for tartrate-resistant acid phosphatase (**G–I**) of hairy lymphocytes and peripheral blood of the same HCL patient in a smear (**D,G**), on anti-CD45 microarray spot (**E,H**) and on anti-CD11c microarray spot (**F,I**). The leukemic cells are morphologically and cytochemically identical; anti-CD11c-captured cells are a pure hairy cell population. Original magnification ×1000 for panels D-I.

10 ml of blood was collected by venipuncture into EDTA-containing tubes and processed within 12 hours from collection. The peripheral blood mononuclear cell (PBMC) fraction was purified using Histopaque 1077 (Sigma) according to manufacturer’s instructions, resuspended in PBS with 1% BSA and 20% of fetal calf serum (Sigma) at 5–8·10^6^ cells/ml concentration, placed on precooled microarray and incubated for 30 min at 4 ^°^C without mixing. Leukocyte concentration of 5–8·10^6^ cells/ml was chosen based on a titration (Fig. 2F) and the incubation at 4 ^°^C was used to avoid nonspecific monocyte binding in the antibody spots (Fig. 2E). The microarray was then washed in PBS containing 1% BSA, then in PBS, placed into the centre of a cytocentrifuge holder, covered with 20 μl of pure fetal calf serum and by rotating around the axis normal to its surface at 5000–6000 rpm. For the analysis of the bone marrow aspirate the mononuclear cell fraction was purified and analysed on the microarray as described above except that the cell concentration in the suspension was 3–5·10^6^ cells/ml and the incubation temperature used was 7 ^°^C.

Lymph node and gastric biopsy specimens were homogenised using manual Dounce tissue grinder with 1 ml capacity (GPE Scientific Limited) in 1% BSA in PBS, washed once, resuspended in 1 ml of PBS with 1% BSA and 20% of fetal calf serum and analysed on the microarray at 4 ^°^C as described above. Spleen samples were sliced with a razor blade, homogenised in Dounce tissue grinder, diluted in PBS containing 1% BSA and layered on Histopaque 1077 for PBMC isolation. The isolated PBMC fraction was analysed at 4 ^°^C as described above. Pleural effusion was centrifuged at 900 g for 3 min to concentrate the cells, the pellet was diluted in PBS with 1% BSA and 20% of fetal calf serum to 4·10^6^ cells/ml and analysed as above.

### Microscopy

The microarray-bound cells were stained after May-Grünwald-Giemsa and examined using Olympus BX45 microscope at ×1000 magnification. Digital pictures were taken with Moticam 1.3 Mpx camera and analysed in ImageJ software. At least 200 captured cells were analysed for each antibody. For determination of cell density the total amount of cells in 3 pictures at ×200 magnification was counted using ImageJ and averaged. The positive control images (anti-CD45 antibody) contained about 1000 cells per image. The standard deviation of the cell density did not exceed 5%.

### Cytochemistry

Acid phosphatase activity was assayed according to Goldberg and Barka^42^. Sudan black B stain was performed using Kit Noir Soudan (Reactifs RAL, France) according to manufacturers’ instructions. α-naphtyl acetate esterase was assayed using Diahim-Cytostain-NE kit (Abris, Russia) according to manufacturers’ instructions except that the reaction was performed for 60 min at room temperature.

### Flow cytometry

For the flow cytometry controls on normal peripheral blood the PBMC were isolated from the whole blood as described above, resuspended in PBS with 1% BSA to the final concentration of 10^7^ cells/ml and incubated with anti-CD4-FITC (MT310, Dako)/anti-CD19-RPE (HD37, Dako)/anti-CD45-PerCP-Cy5.5 (2D1, BD Biosciences) or anti-CD3-FITC (UCHT1, Dako)/anti-CD8-RPE (DK25, Dako)/anti-CD45-PerCP-Cy5.5 (2D1, BD Biosciences) for 20 min at 4 ^°^C. The labelled cells were washed in 2 ml PBS for 3 min at 900 g, resuspended in PBS to final concentration of 1.25·10^6^ cells/ml and analysed on FACSCanto (BD Biosciences, San Jose, CA, USA) flow cytometer. Mononuclear cells were first gated based on their light scatter characteristics and then the percentages of cells double positive for CD45 and CD3, CD4, CD8 or CD19 were determined using FACSDiva 6.1.3 software. The diagnostic flow cytometry for the patients with leukemia and lymphoma was performed on the whole blood using FACSCanto II (BD Biosciences, San Jose, CA, USA) flow cytometer according to current diagnostic guidelines.

### Immunofluorescent specificity analysis

PBMC were isolated from the whole blood as described above and resuspended in PBS with 1% BSA to final concentration of 1.5–2·10^7^ cells/ml. Anti-CD3-FITC (UCHT1, Dako), anti-CD4-RPE (SK3, BD Biosciences), anti-CD8-RPE (DK25, Dako) or anti-CD19-RPE (HD37, Dako) were added to the cell suspension (3 μl per million cells) and incubated at 4 ^°^C for 30 min. In control samples the anti-CD antibodies were replaced by the isotype controls with the same label. The labelled cells from the experimental and control samples were washed in 2 ml PBS for 3 min at 900 g, resuspended in PBS containing 1% BSA and 50% fetal bovine serum to final concentration of 1.25·10^6^ cells/ml. 300 microliters of the cell suspensions were placed on identical precooled microarrays and incubated at 4 ^°^C for 30 min, washed in PBS with 1% BSA, then in PBS, covered with a microscope cover glass and sealed by nail polish.

The DIC and fluorescent images of both samples were acquired with an Axio Observer.Z1 microscope (Carl Zeiss, Jena, Germany) at ×200 magnification. The fluorescent images were analysed using ImageJ software.

Statistical data in the text are presented as average ± s.e.m.

## Additional Information

**How to cite this article**: Khvastunova, A.N. *et al.* Anti-CD antibody microarray for human leukocyte morphology examination allows analyzing rare cell populations and suggesting preliminary diagnosis in leukemia. *Sci. Rep.* **5**, 12573; doi: 10.1038/srep12573 (2015).

## Supplementary Material

Supplementary Information

## Figures and Tables

**Table 1:** Specificity of the lymphocyte binding to the microarray.

Antibody	CD3-FITC	CD4-RPE	CD8-RPE	CD19-RPE
CD3	97 ± 4			2 ± 1
CD4	96 ± 3	97 ± 1	2 ± 2	1 ± 1
CD8	95 ± 3	1 ± 2	98 ± 2	0
CD19	0	1 ± 1	0	93 ± 7

The percentage of the normal PBMC stained positive by the fluorescently labelled antibody marked in the top line from the total amount of cells captured by an antibody against a CD marked in the leftmost column. Every number is an average of two separate experiments. All the cells, positive for CD4 or CD8, also express the pan-T-cell antigen CD3 but not the CD19 typical for the B-cells. The peripheral blood T lymphocytes are positive either for CD4 or CD8. Thus all the cells, captured by anti-CD3, anti-CD4, anti-CD8 and anti-CD19 antibodies, divide into 3 non-overlapping groups, CD3+CD4+, CD3+CD8+ and CD19+ cells, and cover the majority of the peripheral blood lymphocytes.

**Table 2:** Morphological groups of PBMC and their predominant immunophenotype.

	Small lymphocytes	Large lymphocytes		Granular lymphocytes		Monocytes
		Total	Lymphocytes, lobed nucleus	Total	Granular lymphocytes, lobed nucleus	
CD3	93 ± 0.7	**2.5** ± **0.2**	0.8 ± 0.1	4.5 ± 0.7	0.6 ± 0.1	0
CD4	90 ± 1.4	1.9 ± 0.2	0.3 ± 0.1	0.1 ± 0.1	0.3 ± 0.1	8 ± 1
CD8	74 ± 2	2 ± 0.3	**1.5** ± **0.3**	**23** ± **2**	**1.4** ± **0.4**	0
CD16	23 ± 2	4 ± 1	**2.2** ± **0.9**	**54** ± **3**	**2.0** ± **1.0**	19 ± 2
CD19	95.3 ± 0.5	**4.7** ± **0.4**	0*	0*	0*	0
CD45	78 ± 1	2.5 ± 0.3	1.3 ± 0.2	6 ± 1	1.0 ± 0.3	13 ± 1
CD56	33 ± 3	2.0 ± 0.3	**3.5** ± **1.7**	**65** ± **3**	**4.7** ± **1.1**	0.1 ± 0.1
CD14	**0.6** ± **0.1**	0	0	0	**0**	**99.4** ± **0.1**

The numbers represent the percentage of the cells of certain morphology among the mononuclear cells captured by corresponding anti-CD antibodies (average ± s.e.m.) for 40 healthy donors. The numbers, marked with an asterix, are based on an additional study of 2000 of anti-CD19-captured cells for each of 3 healthy donors.
