# Supplementary material for: Anti-CD antibody microarray for human leukocyte morphology examination allows analyzing rare cell populations and suggesting preliminary diagnosis in leukemia
Source: Sci Rep. 2015 Jul 27;5:12573. doi: 10.1038/srep12573 (PMC4515824; doi:10.1038/srep12573)
Supplement: Supplementary Information [file srep12573-s1.pdf]

Supplementary information for

Anti-CD antibody microarray for human leukocyte morphology examination allows analyzing rare cell populations and suggesting preliminary diagnosis in leukemia

Alina N. Khvastunova[1,2], Sofya A. Kuznetsova[1,2,3], Ljubov S. Al-Radi[3], Alexandra V. Vylegzhanina[3], Anna O. Zakirova[1,2], Olga S. Fedyanina[2], Alexander V. Filatov[4], Ivan A. Vorobjev[5], Fazly Ataullakhanov*[1,2,3]

[1]Centre for Pediatric Hematology, Oncology and Immunology, Moscow, Russia;

[2]Centre for Theoretical Problems of Physicochemical Pharmacology RAS, Moscow, Russia;

[3]Research Centre for Hematology, Moscow, Russia;

[4]Institute of Immunology, Moscow, Russia;

[5]A.N. Belozersky Institute and Department of Cell Biology and Histology, Faculty of Biology, M.V. Lomonosov Moscow State University, Moscow, Russia

Corresponding author: Fazly Ataullakhanov, Centre for Pediatric Hematology, Oncology and Immunology, Samory Mashela 1, 117997, Moscow, Russia, e-mail: ataullakhanov.fazly@gmail.com

Table S1. The predominant immunophenotype of the leukemic cells in hairy cell leukemia (HCL) and splenic marginal zone lymphoma (SMZL). The numbers represent the total number of patients with leukemic cells captured by the antibody against the corresponding CD antigen and the percentage of these patients from the total number studied (in parentheses).

Antigen	CD2	CD5	CD10	CD11c	CD19	CD20	CD22	CD23	CD25	CD103
HCL hairy cells (22 patients total)	6 (27%)	1 (5%)	3 (14%)	22 (100%)	22 (100%)	22 (100%)	22 (100%)	3 (14%)	22 (100%)	21 (95%)
SMZL large cells (7 patients total)	0	0	0	2 (29%)	7 (100%)	7 (100%)	7 (100%)	0	0	0
SMZL villous cells (seen in 2/7)	0	0	0	0	2 (100%)	1 (50%)	0	0	0	0

Table S2. The immunophenotype of the circulating leukemic cells in chronic lymphocytic leukemia (CLL), mantle cell lymphoma (MCL) and follicular lymphoma (FL) and leukemic cells, isolated from lymph node biopsy samples of diffuse large B-cell lymphoma (DLBCL) patient, pleural effusion of a patient with hairy HCL and spleen samples from 3 patients with hairy cell leukemia variant (HCL-V). The numbers represent the total number of patients with leukemic cells captured by the antibody against the corresponding CD antigen and the percentage of these patients from the total number studied (in parentheses).

Antigen	CD2	CD5	CD10	CD11c	CD19	CD20	CD22	CD23	CD25	CD103
CLL (37 patients)	2 (5%)	37 (100%)	0	1 (3%)	37 (100%)	37 (100%)	37 (100%)	37 (100%)	22 (59%)	0
MCL (2 patients)	0	2 (100%)	0	0	2 (100%)	2 (100%)	2 (100%)	0	0	0
FL (1 patients)	0	0	1 (100%)	0	1 (100%)	1 (100%)	1 (100%)	0	0	0
Pleural effusion HCL (1 patient)	0	0	0	1 (100%)	1 (100%)	1 (100%)	1 (100%)	0	1 (100%)	1 (100%)
Lymph node DLBCL (2 patients)	0	0	0	0	2 100%	2 (100%)	2 (100%)	0	0	0
Spleen HCL-V (3 patients)	0	0	0	3 100%	3 100%	3 (100%)	3 (100%)	0	0	2 (67%)

Table S3. The immunophenotype of the circulating leukemic cells in acute myeloid leukemia (AML) M2, multiple myeloma (MM), large granular lymphocytic (LGL) leukemia and leukemic cells, isolated from gastric biopsy samples of a patient with an extranodal T-cell lymphoma. The numbers represent the total number of patients with leukemic cells captured by the antibody against

the corresponding CD antigen and the percentage of these patients from the total number studied (in parentheses). In one MM patient no circulating plasma cells were found in peripheral blood.

Antigen	CD2	CD3	CD5	CD7	CD8	CD19	CD33	CD38	CD56	CD103
AML M2<br>(1 patient)	0	0	0	1<br>(100%)	0	0	1<br>(100%)	1<br>(100%)	0	0
MM<br>(5 patients)	0	0	0	0	0	4<br>(80%)	0	4<br>(80%)	4<br>(80%)	0
Stomach<br>ENKL<br>(1 patient)	1<br>(100%)	1<br>(100%)	0	1<br>(100%)	1<br>(100%)	0	0	1<br>(100%)	1<br>(100%)	1<br>100%
LGLL<br>(2 patients)	2<br>100%	2<br>100%	2<br>100%	2<br>100%	2<br>100%	0	0	1<br>50%	2<br>100%	0

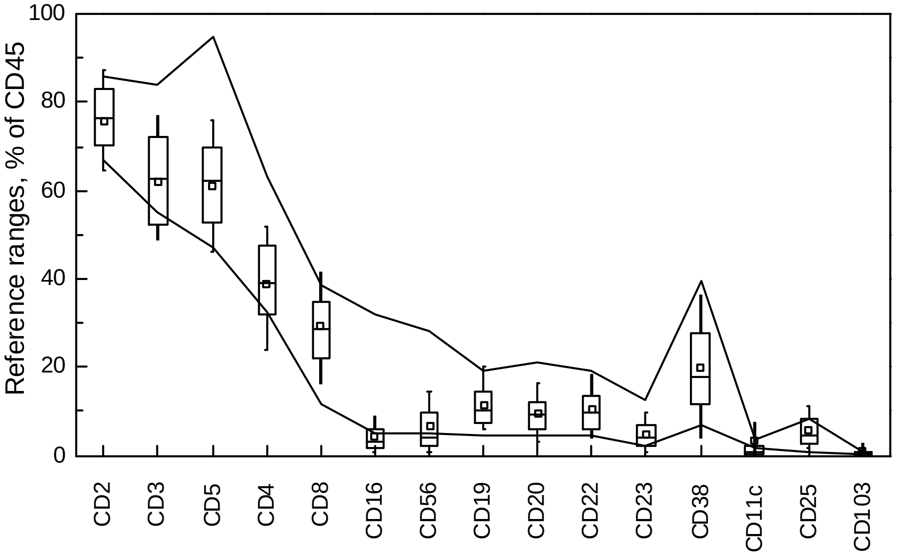

Figure S1. The reference values of normalized cell binding density compared to the reference values for the percentages of lymphocytes positive for the several CD antigens obtained by flow cytometry. The binding density of PBMC from 33 healthy donors captured by different anti-CD, normalized to the cell density on anti-CD45 (white boxes, the boxes indicate the 25%-75% confidence interval, the bars – 5%-95% confidence interval, the middle line indicate the median value, the square – the average value) and the 5%-95% confidence interval for the percentages of lymphocytes positive for the several CD antigens obtained by flow cytometry (lines).

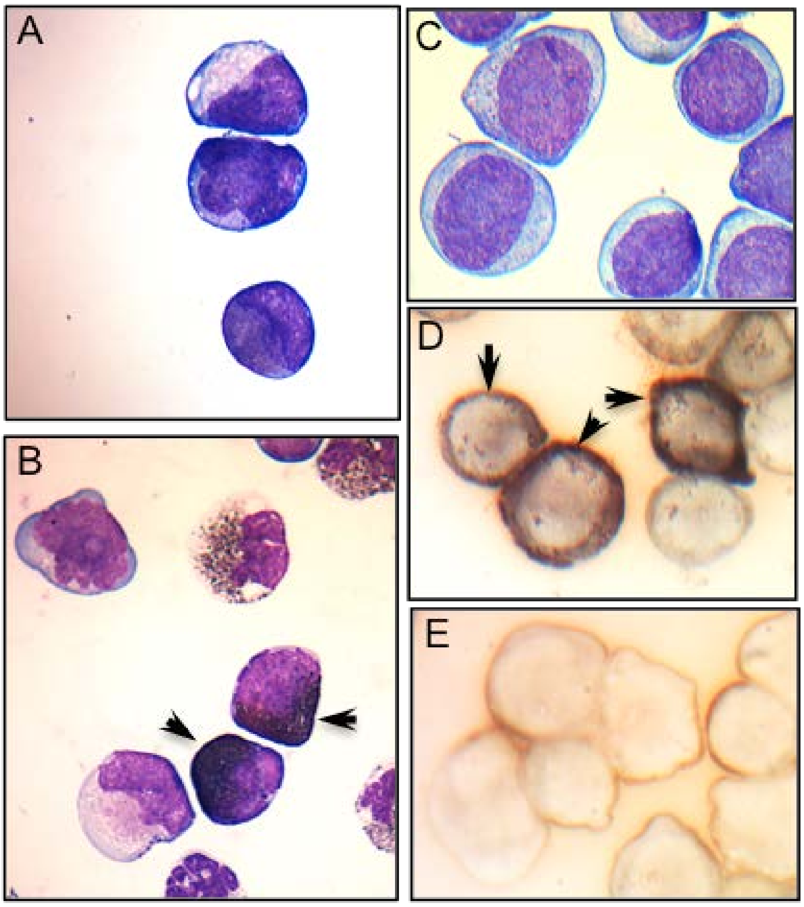

Figure S2. Cytochemical reactions on the microarray. (A, B) Morphology after May-Grünwald-Giemsa stain (A) and Sudan black B reaction (B) for the cells of the bone marrow punctate of a pediatric patient with AML M2 captured by anti-CD13 antibody; (C-E) morphology after May-Grünwald-Giemsa stain (C), nonspecific esterase reaction (α-naphtyl acetate, fast blue B) (D) and nonspecific esterase reaction with NaF inhibition (E) for the cells of the bone marrow punctate of a pediatric patient with AML M5 captured by anti-CD4 antibody. Arrows indicate the neoplastic cells positive for corresponding cytochemistry. Original magnification ×1000 for all panels.

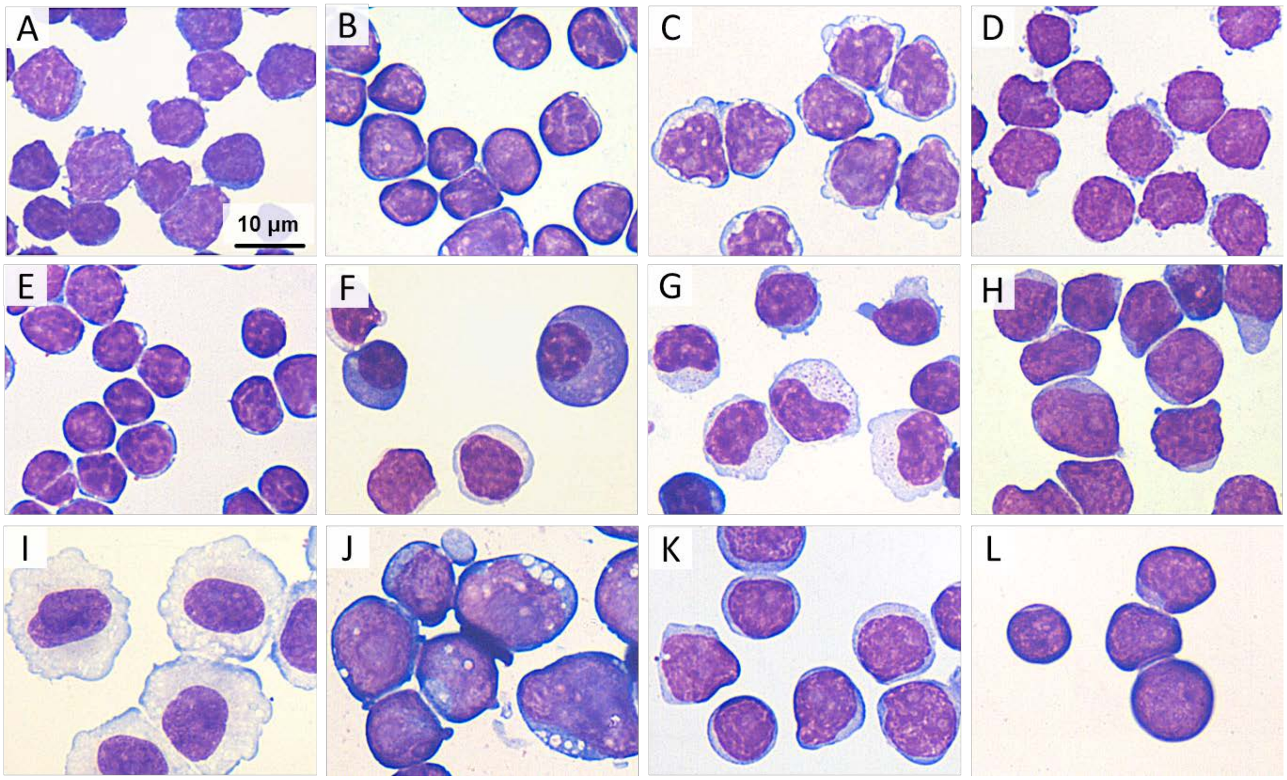

Figure S3. The microarray-captured mononuclear cells from the peripheral blood (A-H), pleural effusion (I), lymph node biopsy (J), spleen (K) and gastric biopsy (L) specimens of the patients with different leukemias and lymphomas after May-Grünwald-Giemsa staining. (A) chronic B-lymphocytic leukemia (B-CLL), peripheral blood, anti-CD19; (B) B-CLL, anti-CD19; (C) splenic marginal zone lymphoma, peripheral blood, anti-CD19; (D) mantle cell lymphoma, peripheral blood, anti-CD19; (E) follicular lymphoma, peripheral blood, anti-CD19; (F) multiple myeloma, peripheral blood, anti-CD38; (G) large granular lymphocytic leukemia, anti-CD8; (H) acute myeloid leukemia M2 (the patient arrived with preliminary diagnosis of hairy cell leukemia), peripheral blood, anti-CD7; (I) hairy cell leukemia, pleural effusion, anti-CD11c; (J) diffuse large B-cell lymphoma, lymph node biopsy, anti-CD19; (K) hairy cell leukemia variant, spleen, anti-CD19; (L) extranodal T-cell lymphoma, gastric biopsy, anti-CD103. Original magnification ×1000.

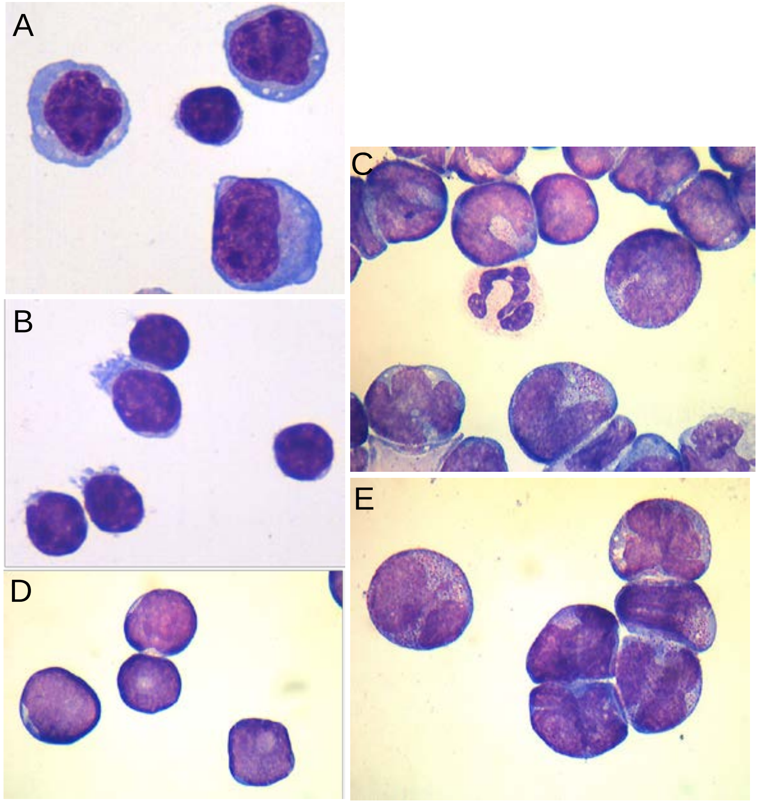

Figure S4. (A-B) Microarray analysis of PBMC from a 57-year-old woman in differential diagnosis between hairy cell leukemia (HCL) and splenic marginal zone lymphoma (SMZL) where morphology and immunohistochemistry data are more consistent with HCL while flow cytometry, cytochemistry and clinical data are characteristic of SMZL. (A) Anti-CD19-captured lymphocytes, leukemic cell 18-25 nm in diameter containing irregular-shaped nuclei with clumped chromatin and light-blue cytoplasm with peripheral basophilia and vacuolization (10% of all anti-CD19-captured PBMC); (B) Anti-CD19-captured lymphocytes, another field of view with small mature lymphocytes with a thin rim of basophilic cytoplasm and thin villi located mostly on one pole of the cell (6% of anti-CD19-captured PBMC). (C-E) Anti-CD microarray permits to separate the two lines of leukemic cells in a bone marrow aspirate of a 7-year-old boy diagnosed with bilineal leukemia (T/myeloid). The microarray analysis of the bone marrow aspirate on the anti-CD protein microarray showed the presence of two morphological groups in the blast population: (i) lymphoid blast cells CD2+, CD3±, CD4+, CD5+, CD21+, CD33±, CD38+, CD45+, CD56+ and CD117+ (C, D) and (ii) myeloid blast cells, positive for CD2+, CD4+, CD7+, CD15+, CD33+, CD38+, CD45+, CD56+, CD64+, CD117+ (C, E). (C) Anti-CD38-captured cells include both lymphoid and myeloid blasts populations; (D) Anti-CD5-captured cells contain only the lymphoid blasts with large nuclear-

cytoplasmic ratio; (E) Anti-CD15-captured cells contain only the large myeloid blasts with irregularly shaped nuclei and granulation.

Data S1. Anti-CD antibody microarray applications in diagnostics.

The study of 22 hairy cell leukemia (HCL) patients and 7 splenic marginal zone lymphoma (SMZL) patients described above as well as our ongoing study of the patients with preliminary diagnosis of HCL permit to formulate the criteria for the microarray-based differential diagnosis of hairy cell leukemia vs hairy cell leukemia variant/splenic red pulp lymphoma and splenic marginal zone lymphoma based of analysis of PBMC binding patterns, morphology and tartrate-resistant acid phosphatase reaction. These results will be fully described in a separate publication. Case 1 illustrates the peripheral mononuclear blood cell analysis for a patient with splenic marginal zone lymphoma using the anti-CD antibody microarray.

Case 1.

A 57 year old woman with 3-year history of normochromic anemia presented to Research centre for hematology for full examination. Physical examination showed no lymphadenopathy, liver or spleen enlargement. The white blood cell (WBC) count of the patient was 6200/μl (band cells – 5%, segmented neutrophils – 32%, basophils – 0%, eosinophils – 1%, monocytes – 5%, lymphocytes – 57%), red blood cells (RBC) – $2.9 \cdot 10^6$/μl, Hb – 85 g/l, platelet count – 54,000/μl. The peripheral blood morphology examination reported 5% of the peripheral blood lymphocytes with vacuolization, diffuse chromatin, some of them with thin villi. Lymphocyte percentage in bone marrow punctate was 59%, trephine biopsy showed diffuse infiltration with widely spaced lymphoid cells of average size with nuclei of irregular shape. The immunohistochemistry of trephine bioptate determined the immunophenotype of the lymphoid infiltrating cells as membrane CD20++, CD25+, membrane annexin+, bcl2+, CD23-, CD5-, CD123-, DBA44-, cyclin D1-, bcl6-. The hairy cell leukemia (HCL) was suggested as the most likely diagnosis based on the infiltration character, CD20, bcl2, annexin and CD25 positivity of the infiltrating cells in trephine bioptate and the hairy cells presence in the bone marrow aspirate, although DBA44, bcl6 and CD123-negativity and normal leukocyte counts are not characteristic of typical HCL. The flow cytometry examination of the peripheral blood detected a clonal CD19+ CD20+ CD23- CD11c- CD103- CD10- B-cell population that was not typical for HCL (CD11c- and CD103-) and suggested a lymphoma of mature B-cells with leukemization, possibly a splenic marginal zone lymphoma (SMZL). The TRAP

reaction in a blood smear was negative and the patient had clonal IgM secretion. The patient was diagnosed with SMZL with circulating villous lymphocytes and treated accordingly.

The examination of the peripheral blood mononuclear cells of this patient on the anti-CD protein microarray showed the increased amount of B-cells (40% of all CD45+ cells was CD19-positive). The amount of CD10, CD11c, CD103 and CD23-positive cells did not differ from the healthy controls in good agreement with the flow cytometry data.

The morphological examination of the anti-CD19-captured cells showed that 84% constituted normal small lymphocytes, 6% - small mature lymphocytes with a thin rim of basophilic cytoplasm and thin villi located mostly on one pole of the cell, 10% - large (18-25 nm in diameter) cells containing irregular-shaped nuclei with clumped chromatin and light-blue cytoplasm with peripheral basophilia and vacuolization (Figure S3 (A-B)). Both the smaller villous and the large pathologic lymphocytes had the same immunophenotype and were captured on the microarray by antibodies to CD19, CD20, CD22, CD25 but not CD23, CD10, CD11c or CD103, showing them to be a single entity. This microarray-derived immunophenotype of the pathologic cells together with the heterogeneity of the leukemic cells and the morphologic characteristics of their subgroups that could not be observed in peripheral blood smears due to their low content (6% of total PBMC) are incompatible with HCL and highly characteristic of SMZL. These data permit to suggest the diagnosis of SMZL directly from the full analysis of the leukemic cell morphology and pattern of their binding to the microarray.

We have analyzed the morphology, cytochemical stains (myeloperoxidase, Sudan black B and nonspecific esterase) and binding patterns of the mononuclear fraction of the bone marrow aspirates of 20 patients with acute B-lymphoblastic leukemia, 2 with T-lymphoblastic leukemia, 35 with acute myeloblastic leukemia and two with acute leukemia of ambiguous lineage and shown that the morphology, cytochemistry and immunophenotype of the neoplastic cells in these cases are in perfect agreement with the flow cytometry, morphology and cytochemistry in smears. The results of this study will be fully described elsewhere. Case 2 illustrates the analysis of neoplastic cells on the anti-CD antibody microarray for the bone marrow aspirate of a patient with acute bilineal leukemia.

Case 2.

A 7 year old boy was admitted to the Centre for pediatric hematology, oncology and immunology with a suspected first bone marrow relapse of acute leukemia. He was treated for acute T-lymphoblastic leukemia (T-ALL), cortical variant, 2 years before and achieved clinical and morphological remission. The white blood cell (WBC) count of the patient on admission was

1000/μl, red blood cells (RBC) – $2.9 \cdot 10^6$/μl, Hb – 124 g/l, platelet count – 41,000/μl. The morphological analysis of the bone marrow punctate reported 72% of undifferentiated blast cells, the blast lineage could not be identified due to considerable cell damage. The flow cytometry examination of the bone marrow leukocytes from the same punctate showed 30% of blast cells (determined by the level of CD45 expression and side scattering (SSC) characteristics) that were positive for surface CD2, CD3 (low expression level), CD4, CD5, CD7, CD21, CD11b, CD15, CD33, CD34, CD38, CD45 (low expression level), CD52, CD65, CD117 and cytoplasmic CD3, CD13 and TdT. The blast population was reported to express T-cell differentiation markers with coexpression of myeloid markers CD15, CD33, CD65 and cytoplasmic CD13, but the levels of myeloid marker coexpression was not estimated due to the presence of other (supposedly normal) myeloid precursors in the analyzed region. The assessment of the bone marrow punctate by cytochemistry in standard smears showed the presence of two blast populations: (i) small cells negative for myeloperoxidase, Sudan black or nonspecific esterase reaction which proves their lymphocyte origin and (ii) myeloid leukemic cells similar to the leukemic cells in acute myeloid leukemia M4 variant by their morphology and the ratio of cells positive for myeloperoxidase and nonspecific esterase. Based on the morphology reassessment and the flow cytometry data the patient was finally diagnosed with acute bilineal leukemia (T/myeloid).

The examination of the leukocyte suspension from the same bone marrow punctate on the anti-CD protein microarray showed the presence of two morphological groups in the blast population. One group (about 20% of all CD45-captured cells) consisted of blast cells of average size with round nuclei, high nuclear-cytoplasmic ratio and no granulation. These lymphoblast-resembling cells were captured by antibodies against CD2, CD3 (only 5% of all anti-CD3-captured cells), CD4, CD5, CD21, CD33 (only 6% of all anti-CD33-captured cells), CD38, CD45, CD56 and CD117. Another population (about 60% of all CD45-captured cells) consisted of large cells with myeloblastic morphology: irregularly shaped nuclei and blue cytoplasm with azurophilic granulation, positive for CD2, CD4, CD7, CD15, CD33 (86% of all anti-CD33-captured cells), CD38, CD45, CD56, CD64, CD117. The anti-CD15 and anti-CD64-captured cells contained exclusively cells of myeloblastic morphology, while anti-CD5 and anti-CD21-captured cells contained only the lymphoblast-like population (Figure S3 (C-E)). The presence of two morphologically distinct populations and the possibility of capturing each of them separately with different anti-CD antibodies clearly indicated a bilineal leukemia.

Such separation of the two leukemic cell lines on different microarray spots permits to study them separately by other methods such as fluorescence in situ hybridizarion and potentially facilitates the control of both cell lines during treatment.
